# Triplane fracture of the proximal tibia: a case report and literature review

**DOI:** 10.11604/pamj.2019.33.40.17953

**Published:** 2019-05-21

**Authors:** Fekih Aymen, Othman Youcef, Saïdi Aymen, Aloui Issam, Abid Abderrazek

**Affiliations:** 1 Department of Trauma and Orthopaedics Surgery, Fattouma Bourguiba University Hospital, Monastir, Tunisia

**Keywords:** Proximal tibial triplane, physeal injury, child

## Abstract

Triplane fractures of the proximal tibia are less well known than the distal extremity. The diagnosis is based on a good analysis of X-rays and possibly CT images to better plan the management. The authors reports a triplane fracture of the proximal tibial in a 12 year old boy treated by closed reduction and internal fixation. To our knowledge, only a dozen cases have been reported in the literature with generally a good evolution of the fracture.

## Introduction

Triplane fracture of the proximal tibia occurs in three planes (transverse, sagittal and coronal) and it typically occurs in adolescence and crosses through the articular surface, the epiphysis, the physis and the metaphysis. Triplane fractures are known in the distal part of the tibia and were first described by Lynn in 1972 [1]. Triplane fractures in the proximal tibia are far less common than those in the distal tibial end, as was demonstrated in the current orthopaedics literature [2-5]. Diagnosis is generally easy with CT scan and treatment is most often surgical, especially in displaced fractures. In this report we describe a triplane fracture of the proximal tibial treated by closed reduction and internal fixation with good result at the last follow-up.

## Patient and observation

**Figure 1:**
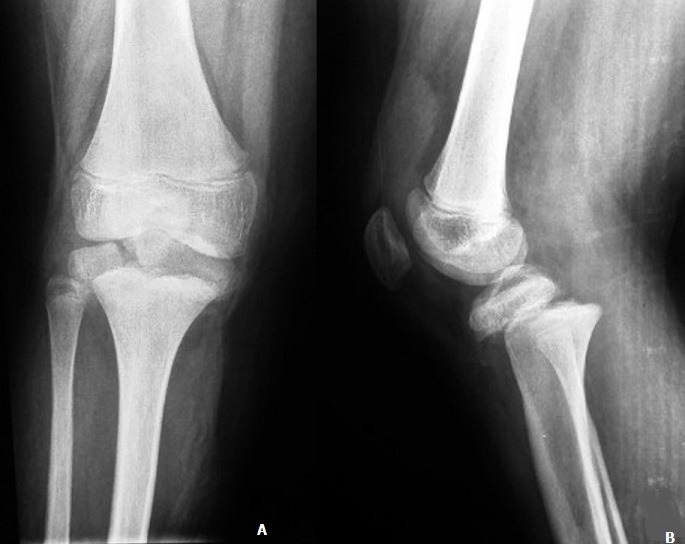
(A) anteroposterior radiograph of the right knee demonstrating Salter-Harris III fracture fragment; (B) lateral radiograph of the right knee demonstrating Salter-Harris I fracture fragment and anterior displacement of the epiphysis

**Figure 2:**
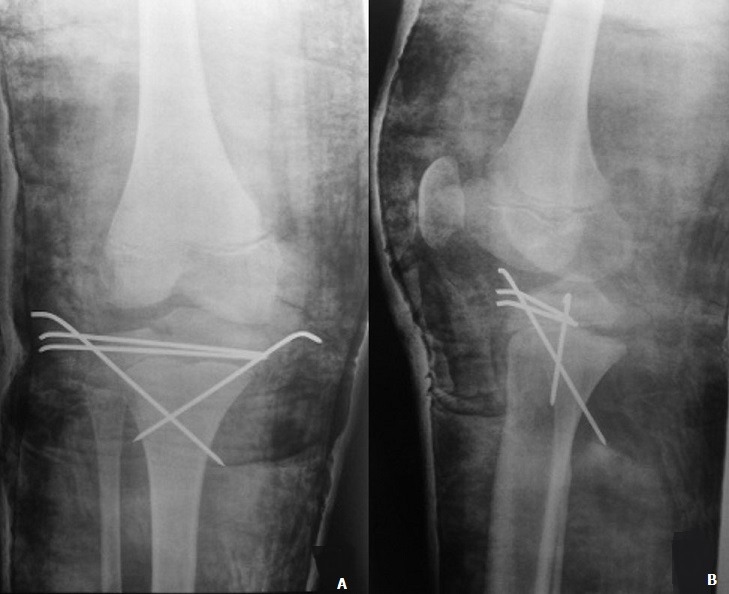
(A) post-operative anteroposterior and lateral (B) radiographs of the right knee

**Figure 3:**
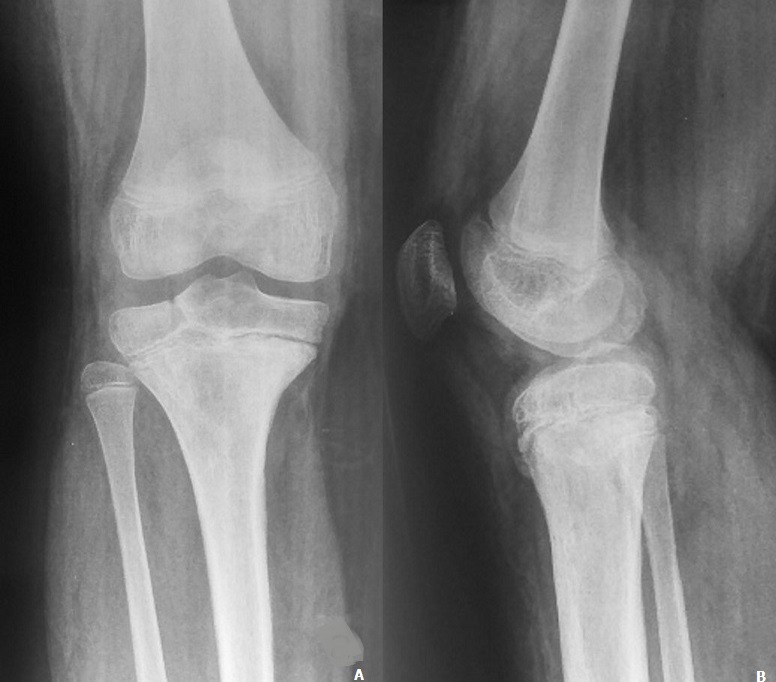
(A) anteroposterior and lateral (B) radiographs of the right knee with consolidation of the fracture at the last one year follow up

**Figure 4:**
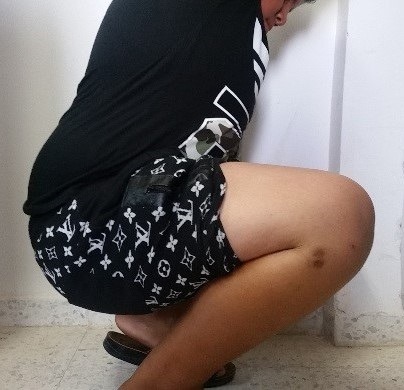
Normal flexion of the right knee at the last one year follow up

A 12-year-old boy with an overweight, consulted the emergency department after a closed trauma of the right knee following a fall from its own height. On examination, he has a painful and swollen knee with limited motion and there was no neurovascular abnormality. The antero-posterior radiograph revealed a fracture through the medial physis extending through the epiphysis classified Salter-Harris III (Figure 1 A). However, the lateral radiograph showed angulation and anterior displacement of the epiphysis classified Salter-Harris I (Figure 1B). Unfortunately, the CT scan was not available that day, so we decided to operate the patient in emergency. Closed reduction was achieved under general anesthesia. The stabilization of the fracture was ensured by a horizontal pinning of the epiphysis and a cross pinning fixing the epiphysis to the metaphysis. A cast immobilization of 6 weeks was necessary to avoid any secondary displacement and to ensure a good consolidation of the fracture (Figure 2). After the removal of the pins, a functional rehabilitation was instituted to regain a good mobility of the knee. At the last one year follow up, the patient had no growth disturbance from injury (Figure 3) and had returned to normal activities with a good mobility of his knee (Figure 4).

## Discussion

Although the fractures of the epiphyseal cartilage injuries are common in the childhood, epiphyseal fractures involving the proximal tibia entities are very rare and account for 1 to 3% of all physeal injuries [6, 7]. Salter-Harris type I to V occur from both indirect and direct mechanism of injury [8]. In distal tibia, the triplane fracture occurs due to the asymmetrical closure of the physis, from central to antero-medial to posteromedial and finishing with closure of the lateral margin of the physis. The proximal tibial physis has a more symmetrical closure, which may explain the rarity of a triplane fracture in this location [5]. Another theory in favor of this rare situation is related to the attachments of the medial and lateral collateral ligaments which result in the stresses from the knee being transmitted to the metaphysis and not the epiphysis [3]. That's why the mostly triplane fractures consist of a coronal fracture of the metaphysis, a transverse fracture of the physis and a sagittal intra-articular fracture of the epiphysis. In practical terms, most triplane fracture are easily diagnosed by plain film radiography. The value of Computed Tomography(CT) especially 3D reconstruction for defining the injury pattern and preoperative planning in these rare and technically demanding cases is highlighted [3, 5]. In triplane fractures involving the proximal tibia, the Magnetic Resonance Imaging (MRI) scan has probably more significant value than the CT scan, as tibial plateau fractures may be associated with cruciate ligament and meniscal tears [9]. We did not perform neither CT scan nor MRI scan because the diagnosis is obvious and imaging was not available that day. The primary goals of treatment are anatomic reduction of the articular fragments with avoidance of further injury to the growth plate. Fracture stabilization is ensured either by screws or by pins. According to some authors [9, 10] the control of the reduction can be performed by knee arthroscopy. The advantages of this arthroscopic reduction are complete evacuation of hemarthrosis, diagnostic evaluation of the cruciate ligaments, the meniscus and the metaphyseal part of the fracture lines extend through the physis into the epiphysis and the joint [4, 5]. A cast immobilization is recommended for at last 6 weeks to prevent secondary displacement and obtain a good consolidation.

## Conclusion

Proximal tibial triplane fractures are rare injuries but carry with them the need for clinical vigilance. These fractures are high energy injuries that can result in multiple intra-articular and physeal fragments that require individual anatomic reduction and stabilization in order to prevent future arthritis, pain, and deformity. Because of the involvement of the physeal growth plate, long-term radiographic follow-up of the affected extremity is necessary to treat any angular deformity or leg length difference that might occur.

## Competing interests

The authors declare no competing interests.
